# Dendritic Cells in Subcutaneous and Epicardial Adipose Tissue of Subjects with Type 2 Diabetes, Obesity, and Coronary Artery Disease

**DOI:** 10.1155/2019/5481725

**Published:** 2019-05-09

**Authors:** Miloš Mráz, Anna Cinkajzlová, Jana Kloučková, Zdeňka Lacinová, Helena Kratochvílová, Michal Lipš, Michal Pořízka, Petr Kopecký, Jaroslav Lindner, Tomáš Kotulák, Ivan Netuka, Martin Haluzík

**Affiliations:** ^1^Department of Diabetes, Diabetes Centre, Institute for Clinical and Experimental Medicine, Prague, Czech Republic; ^2^Department of Medical Biochemistry and Laboratory Diagnostics, First Faculty of Medicine, Charles University and General University Hospital, Prague, Czech Republic; ^3^Centre for Experimental Medicine, Institute for Clinical and Experimental Medicine, Prague, Czech Republic; ^4^Department of Anaesthesiology, Resuscitation and Intensive Medicine, First Faculty of Medicine, Charles University and General University Hospital, Prague, Czech Republic; ^5^2nd Department of Surgery-Department of Cardiovascular Surgery, Charles University and General University Hospital, Prague, Czech Republic; ^6^Anesthesiology Department, Cardiac Centre, Institute for Clinical and Experimental Medicine, Prague, Czech Republic; ^7^Cardiovascular Surgery Department, Cardiac Centre, Institute for Clinical and Experimental Medicine, Prague, Czech Republic

## Abstract

Dendritic cells (DCs) are professional antigen-presenting cells contributing to regulation of lymphocyte immune response. DCs are divided into two subtypes: CD11c-positive conventional or myeloid (cDCs) and CD123-positive plasmacytoid (pDCs) DCs. The aim of the study was to assess DCs (HLA-DR+ lineage-) and their subtypes by flow cytometry in peripheral blood and subcutaneous (SAT) and epicardial (EAT) adipose tissue in subjects with (T2DM, *n* = 12) and without (non-T2DM, *n* = 17) type 2 diabetes mellitus undergoing elective cardiac surgery. Subjects with T2DM had higher fasting glycemia (8.6 ± 0.7 vs. 5.8 ± 0.2 mmol/l, *p* < 0.001) and glycated hemoglobin (52.0 ± 3.4 vs. 36.9 ± 1.0 mmol/mol, *p* < 0.001) and tended to have more pronounced inflammation (hsCRP: 9.8 ± 3.1 vs. 5.1 ± 1.9 mg/ml, *p* = 0.177) compared with subjects without T2DM. T2DM was associated with reduced total DCs in SAT (1.57 ± 0.65 vs. 4.45 ± 1.56% for T2DM vs. non-T2DM, *p* = 0.041) with a similar, albeit insignificant, trend in EAT (0.996 ± 0.33 vs. 2.46 ± 0.78% for T2DM vs. non-T2DM, *p* = 0.171). When analyzing DC subsets, no difference in cDCs was seen between any of the studied groups or adipose tissue pools. In contrast, pDCs were increased in both SAT (13.5 ± 2.0 vs. 4.6 ± 1.9% of DC cells, *p* = 0.005) and EAT (29.1 ± 8.7 vs. 8.4 ± 2.4% of DC, *p* = 0.045) of T2DM relative to non-T2DM subjects as well as in EAT of the T2DM group compared with corresponding SAT (29.1 ± 8.7 vs. 13.5 ± 2.0% of DC, *p* = 0.020). Neither obesity nor coronary artery disease (CAD) significantly influenced the number of total, cDC, or pDC in SAT or EAT according to multiple regression analysis. In summary, T2DM decreased the amount of total dendritic cells in subcutaneous adipose tissue and increased plasmacytoid dendritic cells in subcutaneous and even more in epicardial adipose tissue. These findings suggest a potential role of pDCs in the development of T2DM-associated adipose tissue low-grade inflammation.

## 1. Introduction

Dendritic cells (DCs) are professional antigen-presenting cells with the ability to suppress or instigate immune responses and to bridge innate and adaptive immunity [1]. Upon activation by damage- or pathogen-associated signals, immature DCs undergo a maturation process characterized by expression of surface antigens and cytokines important for priming and activation of naïve T cells and initiation of adaptive immune responses [2]. In addition, DCs also play an important role in maintaining immunological tolerance [3]. Two major subtypes of DCs are recognized according to morphology and cell marker expression: CD11c-positive conventional or myeloid DCs (cDCs) involved in the differentiation of CD4+ T helper (Th) cells and CD123-positive plasmacytoid DCs (pDCs) characterized by production of type I interferon, activation of macrophages, and antiviral defense [2, 4, 5]. Except the typical lymphoid organs such as the spleen and lymph nodes, both types are routinely found in a broad spectrum of nonlymphoid tissues including adipose tissue (AT), where they have recently been implied in the development of metabolic inflammation [2, 6, 7].

Chronic low-grade inflammation characterized by increased accumulation of immune cells, especially macrophages and T lymphocytes, in adipose tissue is one of the main mechanisms associating obesity with insulin resistance, type 2 diabetes mellitus (T2DM), and atherosclerosis [8]. DCs with their capability to influence both T cells and macrophages have been suggested as potential initiators of other immune cell recruitment; however, their role in regulating AT inflammation seems dependent on the actual adipose tissue metabolic state. In lean animals, cDCs were shown to promote an anti-inflammatory state and delay the onset of obesity-induced chronic inflammation and insulin resistance [9]. In contrast, long-term overnutrition induced a proinflammatory switch in their phenotype resulting in activation of Th1 and Th17 responses [10, 11]. The presence of DCs was also essential for AT macrophage recruitment and activation, and high-fat diet increased local AT DC content in murine models [12, 13]. Similarly, in humans, DCs were shown to correlate positively with BMI in subcutaneous adipose tissue (SAT) [10].

Despite the emerging data on adipose tissue DCs, little is known about the presence of DCs in other than subcutaneous and abdominal visceral adipose tissue depots. Epicardial adipose tissue (EAT) with its close proximity to coronary arteries has recently been highlighted as an important player in the development of coronary artery disease [14]. To date, only one experimental study on a murine model of acute coronary syndrome has assessed the presence of DCs in EAT, with no data available in humans [15]. Moreover, the influence of fully developed diabetes mellitus on adipose tissue DC count and phenotype has thus far not been addressed. To this end, we performed a flow cytometry analysis of DCs and their subtypes in peripheral blood and subcutaneous and epicardial adipose tissue of subjects with and without type 2 diabetes mellitus undergoing elective cardiac surgery.

## 2. Methods

### 2.1. Study Subjects

Seventeen subjects without T2DM and 12 subjects with T2DM, all undergoing elective cardiac surgery (coronary artery bypass graft implantation and/or valvular surgery), were included into the study. The diagnosis of coronary artery disease was established by presurgical coronarography. T2DM treatment included metformin (9 subjects), dipeptidylpeptidase-4 inhibitors (3 subjects), sulfonylurea derivatives (2 subjects), and insulin therapy with multiple daily injections (1 subject). Written informed consent was signed by each subject prior to inclusion, and the study was approved by the Human Ethics Review Board, First Faculty of Medicine and General University Hospital, Prague, Czech Republic. The study was performed in accordance with the principles of the Declaration of Helsinki as revised in 2008.

### 2.2. Blood and Adipose Tissue Sampling

Blood samples were taken at the beginning of surgery after overnight fasting. Samples were centrifuged for 10 min at 1000x g within 30 min after withdrawal. Serum or plasma aliquots were subsequently stored at -80 °C.

Analogously, 1-2 g of SAT and EAT was obtained at the beginning of surgery immediately after sternotomy. EAT was taken from the anterior interventricular sulcus or the right margin of the heart, and SAT was obtained from the sternotomy site. Freshly collected specimens in PBS buffer (0.01 M PBS, pH 7.4) were used for flow cytometry, and aliquots in RNAlater® solution (Ambion®- Invitrogen, Carlsbad, California, USA) were stored at -80 °C and subsequently used for determination of mRNA expression. Samples for immunohistochemistry were immediately fixed in 4% formaldehyde and processed further.

### 2.3. Hormonal and Biochemical Assays

Serum levels of cytokines were measured by the multiplex assay MILIPLEX® MAP Human Cytokine/Chemokine Magnetic Bead Panel (Merck KGaA, Darmstadt, Germany). Sensitivity for IFN-*γ* was 0.8 pg/ml, for IL-6 0.9 pg/ml, for IL-8 0.4 pg/ml, and for TNF-*α* 0.7 pg/ml. The intra- and interassay variabilities for all analytes were between 5.0 and 15.0%. Serum high-sensitivity C-reactive protein (hsCRP) levels were measured by high-sensitivity ELISA kit (Bender MedSystems, Vienna, Austria) with a sensitivity of 3 pg/ml. Insulin levels were measured by RIA kit (CIS Bio International, Gif-sur-Yvette, France). Sensitivity was 2.0 *μ*IU/ml. The intra- and interassay variabilities for all assays were between 5.0 and 10.0%.

Biochemical parameters were measured, and LDL cholesterol was calculated at the Department of Medical Biochemistry and Laboratory Diagnostics, General University Hospital, Prague, Czech Republic, by standard laboratory methods.

### 2.4. Isolation of Stromal Vascular Fraction from Adipose Tissue and Flow Cytometry

**Figure 1:**
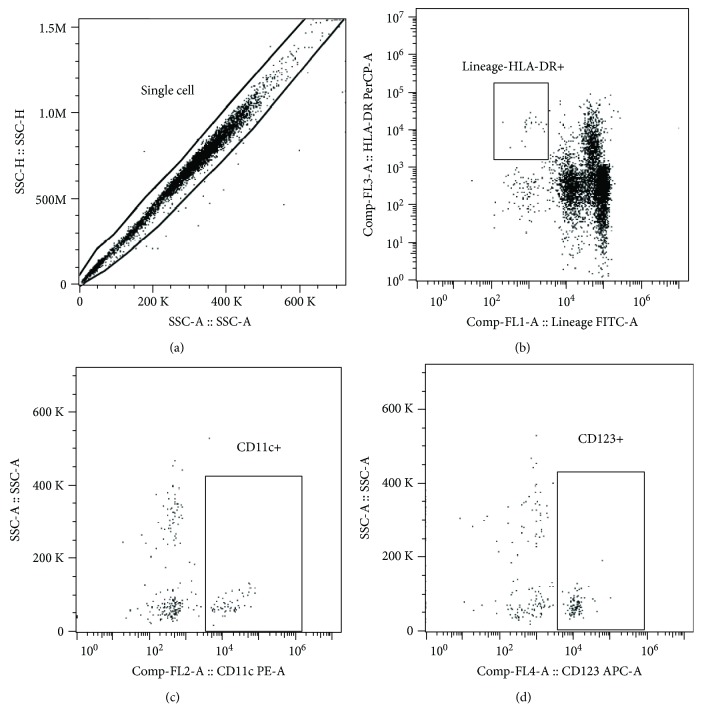
Flow cytometry gating strategy. Gating strategy was as follows: (a) single cells were gated, (b) total dendritic cells were assessed, and (c) CD11c+ conventional dendritic cells or (d) CD123+ plasmacytoid dendritic cells were gated.

Standard 0.5-1.0 g amount of adipose tissue was minced with sterile scissors, and visible blood vessels were removed. Samples were washed with PBS, digested by 0.01% collagenase (Collagenase from Clostridium histolyticum, St. Louis, MO, USA) for 30 min at 37 °C, and centrifuged for 12 min at 1200x g. Visible adipocytes were then manually collected from surface via a pipette with subsequent repeated washings and removal of remaining adipocytes from the supernatant. Finally, samples were filtered through Falcon® 40 *μ*m Cell Strainer (Becton, Dickinson and Company, Franklin Lakes, USA) to eliminate any remnant adipocytes. Flow cytometry was performed using freshly isolated and filtered stromal vascular fraction or EDTA whole blood. A total amount of 100 *μ*l of cell suspension with average 10^6^ cell content was labeled by monoclonal antibodies conjugated with FITC (fluorescein isothiocyanate), PE (phycoerythrin), PerCP (peridinin-chlorophyll protein complex), and APC (allophycocyanin). For labelling a commercial lineage cocktail (CD3/CD14/CD16/CD19/CD20/CD56) FITC and single-labelled antibodies CD11c PE, HLA-DR PerCP, and CD123 APC (Exbio Prague, a.s., Vestec, Czech Republic) were used. The samples were labeled in the dark for 30 min at 2–8 °C, and then red cells were lysed using Excellyse I (Exbio Prague, a.s., Vestec, Czech Republic) according to the manufacturer's instructions. Finally, labelled cells were analyzed on BD Accuri™ C6 (Becton, Dickinson and Company, Franklin Lakes, NJ, USA). Data analysis was performed using FlowJo X 10.0.7r2 software (FlowJo, LCC, Ashland, OR, USA). Gating strategy was as follows: doublets were excluded, dendritic cells were gated according to HLA-DR positivity and lineage cocktail negativity, and then CD11c-positive and CD123-positive cells were assessed (Figure 1). HLA-DR+ lineage DC cells are expressed as percentage of single cells and their subtypes are expressed as percentage of HLA-DR+ lineage- DCs to determine DC composition. Minimal count of acquired events was 100,000.

### 2.5. Statistical Analysis

Statistical analysis was performed and graphs were drawn using SigmaPlot 13.0 (SPSS Inc., Chicago, IL, USA). Results are expressed as mean ± standard error of the mean (SEM). The unpaired *t*-test or Mann-Whitney rank sum test and paired *t*-test or Wilcoxon signed-rank test were used for the assessment of intergroup differences, as appropriate. The Spearman or Pearson correlation test was used to assess the association between DCs and other measured parameters. Multiple linear regression was used to assess the influence of T2DM, obesity, and coronary artery disease on the presence of DCs in tissues. Baseline data of all study subjects were used for correlation and regression analyses. Statistical significance was assigned to *p* < 0.05.

## 3. Results

### 3.1. Anthropometric and Biochemical Parameters

As expected, subjects with T2DM had higher fasting glucose and glycated hemoglobin levels along with increased BMI (body mass index) relative to non-T2DM individuals. Other anthropometric and biochemical parameters including age, lipid profile, creatinine, and hsCRP were comparable between both groups (Table 1). T2DM subjects showed elevated circulating levels of TNF-*α* and MCP-1 relative to the non-T2DM group, while no difference was present in other measured cytokines (Table 2).

### 3.2. Dendritic Cells in Peripheral Blood, SAT, and EAT

**Figure 2:**
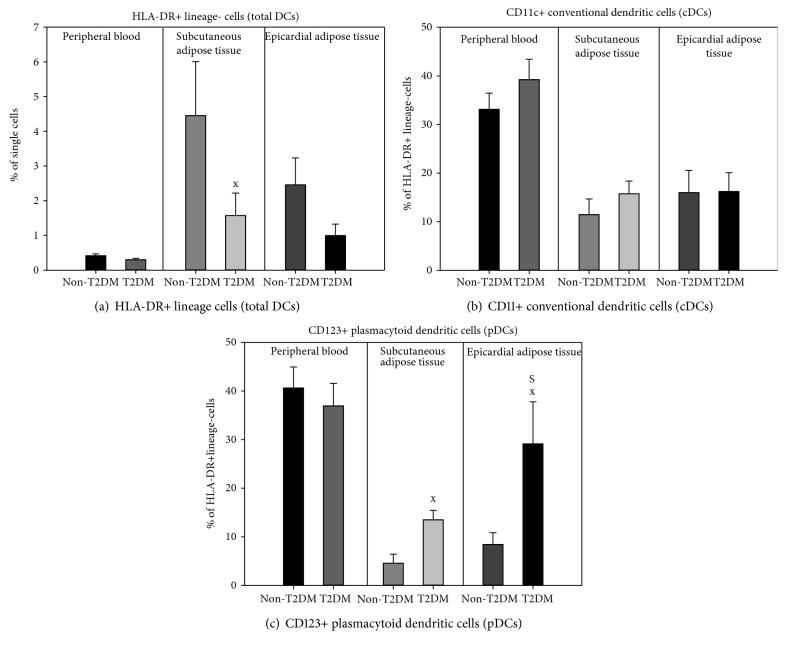
Dendritic cell populations in peripheral blood and subcutaneous and epicardial adipose tissue in subjects with and without type 2 diabetes mellitus. T2DM: type 2 diabetes mellitus; DCs: dendritic cells. Total DCs are gated as percentage of single cells, and other populations are gated as percentage of total DCs. ^x^*p* < 0.05 T2DM vs. non-T2DM; ^S^*p* < 0.05 subcutaneous vs. epicardial adipose tissue.

The total number of circulating DCs and their subtypes showed no differences between both study groups (Figure 2). T2DM subjects had reduced total DCs in SAT (1.57 ± 0.65 vs. 4.45 ± 1.56% for T2DM vs. non-T2DM, *p* = 0.041), while showing a similar, though insignificant, tendency in EAT (0.996 ± 0.33 vs. 2.46 ± 0.78% for T2DM vs. non-T2DM, *p* = 0.171). In either group, the amount of total DCs was comparable between SAT and EAT (Figure 2(a)). When analyzing DC subsets, no difference in the percentage of cDC could be seen between any of the studied groups or adipose tissue pools (SAT: 15.7 ± 2.6 vs. 11.5 ± 3.2% for T2DM vs. non-T2DM, *p* = 0.335; EAT: 16.2 ± 3.9 vs. 16.0 ± 4.6% for T2DM vs. non-T2DM, *p* = 0.724) (Figure 2(b)). In contrast, pDCs were increased in both SAT (13.5 ± 2.0 vs. 4.6 ± 1.9% of DC cells, *p* = 0.005) and EAT (29.1 ± 8.7 vs. 8.4 ± 2.4% of DC, *p* = 0.045) of T2DM relative to non-T2DM subjects as well as in EAT of the T2DM group compared with corresponding SAT (29.1 ± 8.7 vs. 13.5 ± 2.0% of DC, *p* = 0.020) (Figure 2(c)).

### 3.3. Association of Dendritic Cells with Other Measured Parameters

Total circulating DCs inversely correlated with glycemia (*R* = −0.528, *p* = 0.004) and hsCRP (*R* = −0.399, *p* = 0.048). Multiple linear regression showed glycemia as the only independent predictor (*p* = 0.044, Adj Rsqr = 0.129). Circulating cDCs positively correlated with serum IFN-*γ* (*R* = 0.425, *p* = 0.043) and inversely with C-peptide (*R* = −0.486, *p* = 0.019) and hsCRP levels (*R* = −0.434, *p* = 0.030), with only C-peptide exerting an independent association (*p* = 0.012, Adj Rsqr = 0.262). No correlation of total DCs, cDCs, or pDCs with any assessed parameters was observed in either SAT or EAT.

Multiple linear regression showed that the prevalence of DCs in EAT was associated with T2DM (*p* = 0.015), but not with obesity (*p* = 0.051) or coronary artery disease (*p* = 0.091), the Adj Rsqr = 0.424. Similarly, T2DM was the sole positive predictor of pDCs in SAT (*p* = 0.025, Adj Rsqr = 0.398) as well as in EAT (*p* = 0.036 for T2DM, Adj Rsqr = 0.377) with neither obesity nor coronary artery disease showing any association in either adipose tissue depot. In contrast, cDC showed no correlation with either T2DM, obesity, or CAD in any studied compartment.

## 4. Discussion

Dendritic cells are professional antigen-presenting cells responsible for maintaining immunological tolerance and coupling innate and adaptive immunity [7]. Adipose tissue DCs have been suggested to contribute to adipose tissue inflammation by regulating macrophage and T cell accumulation and activation [2]. Here, we show that in subjects with T2DM, total DCs are decreased in subcutaneous adipose tissue compared with nondiabetic individuals, while the subset of plasmacytoid DCs is increased both in SAT and EAT as well as in EAT relative to SAT.

Compared with the evidence of positive association between obesity and high-fat feeding and increased number of AT DCs, data on the relationship between AT DCs and insulin resistance or type 2 diabetes mellitus are much scarcer. Bertola et al. [10] showed positive correlation between DC marker CD1c and HOMA index of insulin resistance in SAT of subjects with and without T2DM and obesity. In a murine model, Cho et al. [13] demonstrated that the absence of the chemokine receptor CCR7, which is necessary for the high-fat diet-induced accumulation of DCs in adipose tissue, was associated with lower fasting glucose and insulin levels. In another experimental study, mice completely lacking DCs were protected against diet-induced obesity and insulin resistance; however, in this model, DCs were absent not only from adipose tissue but also from all other organs including the liver, which may have also contributed to the outcomes [12]. Here, we have rather surprisingly found reduced numbers of total DCs in SAT of T2DM subjects with a similar trend observed also in EAT despite increased fasting glucose and higher BMI in the diabetic group. On the other hand, higher prevalence of total DCs in SAT relative to EAT in both groups corresponds well with the findings of Cho et al. [13], who reported increased numbers of DCs in SAT compared with omental adipose tissue in obese humans undergoing bariatric surgery. The partial discrepancies with previous results are not easily explained; however, as this is the first human study to directly assess the number of DCs in diabetic vs. nondiabetic subjects, no directly comparable data to verify these findings are available to date. Nevertheless, differences in DC assessment methodology and limited transferability of animal data to humans might have contributed to these outcomes. Clearly, other studies on larger patient populations are needed to clarify this issue.

Interestingly, while conventional DCs did not differ between both groups, plasmacytoid DCs were increased in diabetic subjects in both SAT and EAT in a manner completely opposite to total DCs. These findings are in line with the data of Ghosh et al. [16] who showed a positive correlation between the expression of pDC-specific transcripts (CLEC4C, INF signature genes) and HOMA index of insulin resistance in visceral adipose tissue of obese diabetic subjects. They also confirm the results of a murine model deficient in pDCs, in which the animals were protected from diet-induced obesity and insulin resistance [17]. Our findings thus suggest that in human adipose tissue, pDCs might be more important for the development of insulin resistance and T2DM than total or conventional DCs.

Epicardial adipose tissue, localized predominantly in the atrioventricular and interventricular grooves and free wall of the right ventricle, has been shown to be more proinflammatory than SAT and, due to its proximity to coronary arteries and the absence of a dividing fascia, has been suggested to directly contribute to the development of coronary artery disease [18–21]. Even though human data are rather limited, especially compared with SAT and abdominal VAT, EAT was reported to harbor most types of immune cells including macrophages, different subsets of T lymphocytes, and natural killer (NK) cells and B lymphocytes [15, 22, 23]. However, to date, only minimal information on DCs in animal EAT has been available, while no data exist for humans. In a model of acute coronary syndrome, Horckmans et al. [15] showed that acute ligation of coronary arteries was associated with increased amount of DCs in EAT due to their migration from the infarct site in the myocardium. This process was reversed by B lymphocyte depletion or neutralization of granulocyte-macrophage colony-stimulating factor (GM-CSF). Similarly, in obese diabetic db/db mice, DCs accumulated predominantly in perivascular adipose tissue as compared with vessel wall and total depletion of DCs improved vasorelaxation in mesenteric arteries [24]. Here, we for the first time in humans identify total as well as conventional and plasmacytoid DCs in EAT. Strikingly, while total DCs tended to be lower in EAT relative to corresponding SAT regardless of the presence of T2DM as well as in EAT of diabetic compared with nondiabetic subjects and cDCs did not show any meaningful difference between both groups and adipose tissue pools, pDCs were significantly increased in EAT of T2DM individuals relative to both corresponding SAT and EAT of nondiabetics. This was corroborated by positive association between EAT pDCs and the presence of T2DM in multiple regression analysis. Interestingly, no independent relationship between EAT pDCs and obesity or coronary artery disease could be established in either group. As already mentioned, pDCs might contribute to adipose tissue inflammation by a number of mechanisms including macrophage recruitment and M1 polarization, maturation of conventional DCs, activation of B and T cells, or altered immune-metabolic interplay resulting from increased type I IFN production [9, 12, 16, 25–28]. Collectively, these data suggest a specific proinflammatory phenotype of EAT mediated by plasmacytoid DCs that is further augmented by the presence of type 2 diabetes mellitus. This hypothesis is supported by a recent transcriptomic analysis showing increased expression of a number of proinflammatory genes involved in TNF-*α*, NF-*κ*B, and other inflammatory pathways in EAT from diabetic relative to nondiabetic individuals [29]. The cross-sectional nature of our study does not enable us to dissect cause from consequence; however, our data warrant further research into the function of pDCs in EAT, as these cells might comprise a potentially interesting target for therapeutic interventions.

The main limitations of our study include the relatively low sample size and preexisting differences, albeit mostly insignificant, in several baseline characteristics between both groups including BMI and the prevalence of coronary artery disease and arterial hypertension, even though multiple regression analysis was performed to account for most of the differences. Two different EAT sampling sites—anterior interventricular sulcus and right cardiac margin—could have also influenced the results as the periventricular and pericoronary location of EAT was recently shown to have different transcriptomic signatures [30]; however, sampling location in our study was guided primarily by the availability of EAT and thus could not be kept absolutely uniform in all participants.

## 5. Conclusions

Taken together, our study demonstrated for the first time in humans the presence of dendritic cells and their both subtypes (conventional and plasmacytoid) in epicardial adipose tissue. It further showed that type 2 diabetes mellitus is associated with the reduction of total DCs in subcutaneous adipose tissue, while pDCs are conversely increased in subcutaneous and even more in epicardial adipose tissue. These findings suggest a potential role of pDCs in the development of T2DM and as a future therapeutic target.

## Figures and Tables

**Table 1:** Baseline characteristics of study subjects.

	Non-T2DM	T2DM
Number of subjects (males/females)	17 (14/3)	12 (9/3)
Age (year)	65.9 ± 3.3	66.2 ± 2.2
BMI (kg/m^2^)	27.4 ± 1.0	32.6 ± 1.2^x^
Fasting glycemia (mmol/l)	5.81 ± 0.24	8.55 ± 0.74^x^
HbA_1c_ (mmol/mol)	36.9 ± 1.0	52.0 ± 3.4^x^
Total cholesterol (mmol/l)	3.85 ± 0.25	4.39 ± 0.24
HDL cholesterol (mmol/l)	1.11 ± 0.07	1.33 ± 0.17
LDL cholesterol (mmol/l)	2.22 ± 0.21	2.34 ± 0.16
Triglycerides (mmol/l)	1.24 ± 0.13	1.79 ± 0.36
hs C-reactive protein (mg/ml)	5.06 ± 1.89	9.83 ± 3.08
Creatinine (*μ*mol/l)	81.2 ± 5.4	82.8 ± 4.8
Arterial hypertension (*n*, %)	14 (82.4%)	12 (100%)
LVEF (%)	56.6 ± 3.9	50.6 ± 5.7
Coronary artery disease (*n*, %)	9 (52.9%)	9 (75.0%)

Data are presented as mean ± SEM. ^x^*p* < 0.05 vs. non-DM. LVEF: left ventricular ejection fraction; CABG: coronary artery bypass graft.

**Table 2:** The influence of T2DM on circulating cytokine levels.

	Non-T2DM (*n* = 17)	T2DM (*n* = 12)
TNF-*α* (pg/ml)	4.224 ± 0.443	8.285 ± 1.439^x^
IFN-*γ* (pg/ml)	6.737 ± 2.232	5.599 ± 1.813
IL-6 (pg/ml)	2.586 ± 1.914	2.663 ± 1.062
IL-8 (pg/ml)	1.732 ± 0.385	3.168 ± 0.758
IL-23 (pg/ml)	453.7 ± 87.3	264.3 ± 67.5
MCP-1 (pg/ml)	121.5 ± 8.6	148.3 ± 26.6

Data are presented as mean ± SEM. ^x^*p* < 0.05 vs. without T2DM.

## Data Availability

The data used to support the findings of this study are available from the corresponding author upon request.
